# Trajectories of health conditions and their associations with the risk of cognitive impairment among older adults: insights from a national prospective cohort study

**DOI:** 10.1186/s12916-024-03245-x

**Published:** 2024-01-10

**Authors:** Min Du, Liyuan Tao, Min Liu, Jue Liu

**Affiliations:** 1 Department of Epidemiology and Biostatistics, School of Public Health, Peking University, Beijing, 100191 China; 2 Research Center of Clinical Epidemiology, Peking University Third Hospital, Beijing, 100191 China; 3 Institute for Global Health and Development, Peking University, Beijing, China; 4 Key Laboratory of Epidemiology of Major Diseases (Peking University), Ministry of Education, Beijing, China; 5 Department of Global Health and Population, Harvard TH Chan School of Public Health, Boston, MA 02115 USA

**Keywords:** Depressive symptoms, Functional limitations, Multimorbidity status, Sleep disturbances, Cognitive impairment

## Abstract

**Background:**

The associations between trajectories of different health conditions and cognitive impairment among older adults were unknown. Our cohort study aimed to investigate the impact of various trajectories, including sleep disturbances, depressive symptoms, functional limitations, and multimorbidity, on the subsequent risk of cognitive impairment.

**Methods:**

We conducted a prospective cohort study by using eight waves of national data from the Health and Retirement Study (HRS 2002–2018), involving 4319 adults aged 60 years or older in the USA. Sleep disturbances and depressive symptoms were measured using the Jenkins Sleep Scale and the Centers for Epidemiologic Research Depression (CES-D) scale, respectively. Functional limitations were assessed using activities of daily living (ADLs) and instrumental activities of daily living (IADLs), respectively. Multimorbidity status was assessed by self-reporting physician-diagnosed diseases. We identified 8-year trajectories at four examinations from 2002 to 2010 using latent class trajectory modeling. We screened participants for cognitive impairment using the 27-point HRS cognitive scale from 2010 to 2018 across four subsequent waves. We calculated hazard ratios (HR) using Cox proportional hazard models.

**Results:**

During 25,914 person-years, 1230 participants developed cognitive impairment. In the fully adjusted model 3, the trajectories of sleep disturbances and ADLs limitations were not associated with the risk of cognitive impairment. Compared to the low trajectory, we found that the increasing trajectory of depressive symptoms (HR = 1.39; 95% CI = 1.17–1.65), the increasing trajectory of IADLs limitations (HR = 1.88; 95% CI = 1.43–2.46), and the high trajectory of multimorbidity status (HR = 1.48; 95% CI = 1.16–1.88) all posed an elevated risk of cognitive impairment. The increasing trajectory of IADLs limitations was associated with a higher risk of cognitive impairment among older adults living in urban areas (HR = 2.30; 95% CI = 1.65–3.21) and those who smoked (HR = 2.77; 95% CI = 1.91–4.02) (all *P* for interaction < 0.05).

**Conclusions:**

The results suggest that tracking trajectories of depressive symptoms, instrumental functioning limitations, and multimorbidity status may be a potential and feasible screening method for identifying older adults at risk of cognitive impairment.

**Supplementary Information:**

The online version contains supplementary material available at 10.1186/s12916-024-03245-x.

## Background

Cognitive impairment is a prevalent condition among older individuals, which manifests as difficulties in memory, learning, concentration, and decision-making. With rapid population aging, the prevalence of cognitive impairment will be higher [1]. Chen et al. reported that the prevalence of mild cognitive impairment in older adults was 21.2% [2]. Cognitive impairment is associated with various negative health outcomes, including an increased risk of mortality, the onset of dementia, higher rates of disability and hospitalization, and a decline in quality of life [3, 4]. Therefore, the prevention and management of cognitive impairment in the elderly have long been crucial concerns in promoting the well-being of the aging population.

As the global population increasingly ages, the coexistence of physical and psychological disorders is emerging as a significant worldwide challenge [5]. Additionally, there may be interactions between physical and psychological disorders. Sleep disorders, depressive symptoms, functional limitations, and multimorbidity each belong to different categories of physical or psychological disorders. Previous studies have reported that those disorders are risk factors that can adversely affect cognitive function [6–9]. However, most of the previous studies only conducted single-point measurements of these variables. Such single assessments of them frequently resulted in inconsistent findings and erroneous associations. Because patients’ sleep disorders and depressive symptoms often fluctuated at different stages of the illness, this could lead to erroneous associations with the risk of cognitive impairment. The remitting and relapsing nature of sleep disorders and depressive symptoms necessitates an investigation into their course in relation to the risk of cognitive impairment. Additionally, sleep disorders, depressive symptoms, and functional limitations were associated with some chronic diseases, such as cardiovascular diseases, which were also related to cognitive impairment [10, 11]. Consequently, extended observation over a longer period could uncover more insightful associations that a single assessment might overlook.

In recent years, there have been limited studies exploring the impact of trajectories of depressive symptoms, functional limitations, and sleep disorders on cognitive function decline. Mirza et al. reported that the increasing depressive symptoms were associated with a higher risk of dementia compared to the low depressive symptom trajectory [12]. Kaup et al. also found similar results, and they also discovered that a moderate and increasing trajectory of depressive symptoms was not significantly associated with the risk of dementia after full adjustment [13]. Yang et al. discovered that among Chinese older individuals, there was a high risk of mild cognitive impairment in the increasing instrumental activities of daily living (IADLs) limitation group, as reported using a group-based trajectory model [14]. Changes in sleep duration over time were also independently associated with cognitive decline among older adults [15, 16]. Due to the interplay of these physical and psychological disorders and their potential relationship with cognitive impairment, it is necessary to use long-term cohort follow-up data to investigate the independent impact of multiple disease trajectories on cognitive impairment, after controlling for other confounding factors.

To address the aforementioned issues, we employed 8 years of repeated measurement data from the Health and Retirement Study (HRS) to examine different trajectories and their subsequent effects on cognitive impairment. Our study aimed to investigate the relationship between long-term health status and cognitive impairment and to provide a reference for disease surveillance and prevention in clinical practice.

## Methods

### Study design and participants

We utilized cohort data from the HRS, a nationally representative longitudinal survey of adults aged 50 years and older in the USA [17, 18]. The HRS collected information on demographics, socioeconomic status, physical and mental health, and more. Further details on the sample design and procedures can be found in the cohort profile [17]. The standardized and validated measures of sleep disturbances, depressive symptoms, and functional limitations ensured the reliability and validity of the HRS study. The reporting of this study conforms to the Strengthening the Reporting of Observational Studies in Epidemiology guidelines.

**Fig. 1:**
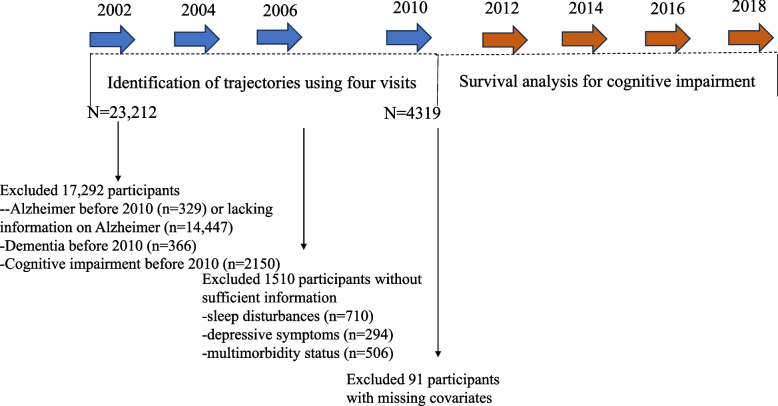
Study flowchart

Our study included participants who were not explicitly diagnosed with cognitive impairment, dementia, or Alzheimer’s disease, but had data on sleep conditions, depressive status, functional limitations, and multimorbidity status at four examination rounds in 2002, 2004, 2006, and 2010. We used this data to identify 8-year trajectories of sleep disturbances, depressive symptoms, functional limitations, and multimorbidity status. Participants were followed up for cognitive impairment until 2018, with assessments conducted every 2 years. This study utilized data from four assessments of the participants to construct trajectory models for different health conditions. At baseline, a total of 23,212 participants aged 60 years or older were included. After excluding 329 participants with Alzheimer’s disease and those who lacked information on Alzheimer’s disease (*n* = 14,447), 366 participants with dementia, and 2150 participants with cognitive impairment before 2010, we further excluded 710 participants without sufficient information on sleep disturbances, 294 participants without sufficient information on depressive symptoms, and 506 participants without sufficient information on multimorbidity status, as well as those with missing information on covariates (*n* = 91). Finally, we included 4319 participants in this cohort study (Fig. 1).

The HRS has received approval from the University of Michigan Institutional Review Board (IRB Protocol: HUM00061128). All participants provided oral informed consent.

### Assessment of sleep disturbances, depressive symptoms, functional limitations and multimorbidity status

Sleep disturbances were assessed using a modified version of the Jenkins Sleep Scale, which demonstrated a good reliability (Cronbach’s alpha) of at least 0.7 [19]. Four items, including difficulty falling asleep, difficulty staying asleep, waking up too early during the night, and feeling rested when waking up in the morning, were considered. Participants were asked to rate their responses on a scale of 1 to 3, with “1 = rarely or never,” “2 = sometimes,” and “3 = most of the time.” After reverse coding the last item, the scores were summed, resulting in a total score ranging from 3 to 12 [20]. A higher score indicated a higher level of sleep disturbances [20].

Depressive symptoms were assessed using the 8-item Centers for Epidemiologic Research Depression (CES-D) scale, which measured the frequency of feelings on eight dichotomous items in the past week, including “depressed,” “everything was an effort,” “happy,” “life was enjoyable,” “sad,” and “unable to get going” [21, 22]. We reverse-coded the items on “happy” and “life was enjoyable” and then summed all the items. The total scores ranged from 0 to 8, with higher scores indicating more severe depressive symptoms.

Functional limitations were assessed by using activities of daily living (ADLs) and IADLs, respectively. ADLs limitations were assessed in six activities of daily living, including walking across a room, dressing, bathing, eating, getting in or out of bed, and using the toilet [23]. IADLs limitations were assessed in five instrumental activities of daily living, which included using a telephone, managing money, taking medications, shopping for groceries, and preparing hot meals [23]. If the respondent reported no difficulty with the item, it was coded as 0. If they reported difficulty, it was coded as 1. ADLs ranged from 0 to 6, and IADLs ranged from 0 to 5, with higher scores indicating greater ADLs limitations and IADLs limitations.

Multimorbidity status was assessed by calculating the sum score of 8 self-reported physician-diagnosed conditions: type 2 diabetes, stroke, hypertension, heart disease, chronic lung disease, arthritis, psychological diseases, and cancer [23].

### Assessment of cognitive impairment

The cognitive function was assessed at each survey wave using the 27-point HRS cognitive scale. This scale consisted of immediate and delayed 10-noun free recall to assess memory, serial sevens subtraction to assess working memory, and counting backwards to assess the speed of mental processing [19, 24]. The total scores ranged from 0 to 27, with a score of 11 or less indicating cognitive impairment [23, 24].

### Covariates

We assessed covariates using information from the 2010 examination. The following measures include age (< 70 years, 70 ~ 79 years, ≥ 80 years), gender (female, male), educational level (less than high school, high school or associate degree, some college or associate degree, college degree or above), marital status (married, unmarried), residence (rural, urban), total wealth income (the lowest quartile, Quartile 2, Quartile 3, the highest quartile), self-reported body mass index (BMI; underweight, normal, obesity, overweight) [25], physical activity (no, yes), drinking (no, yes), and smoking (no, yes) were considered.

### Statistical analysis

We used latent class trajectory models (LCTM) to identify trajectories of sleep disturbances, depressive symptoms, ADLs limitations, IADLs limitations, and multimorbidity status over time [12]. LCTM, as a finite mixture model, can identify latent classes of individuals who exhibit similar progressions of a determinant over time or with age [12]. Our models utilized second-order polynomials and calculated the posterior probabilities for each trajectory, taking into account the age level. Every participant was assigned to the trajectory with the highest probability. The best-fitting number of trajectories was selected based on the minimum Bayesian Information Criterion (BIC), while ensuring the posterior probabilities by class (> 0.70) and class size (≥ 2% of the population). To facilitate interpretability, we assigned labels to the trajectories based on the basis of their modeled graphic patterns.

To assess the risk of cognitive impairment, the examination date of the fourth examination (2010) was considered as time zero for the survival model. Time to event (cognitive impairment) was defined as follows: participants were followed up from the start date of the survival analyses (2010) and censored on the date of cognitive impairment, death, or being lost to follow-up. Participants were censored on the date that they were last seen or contacted when they were lost to follow-up. After assessing adherence to the proportional hazards assumption by plotting smoothed Schoenfeld residuals against time, there were no violations of the assumption. The hazard ratio (HR) for cognitive impairment was computed by using Cox proportional hazard models, based on the assigned trajectory. For all analyses, we fitted three models: model 1 was a univariate model; model 2 adjusted for age, gender, educational level, marital status, residence, total wealth income, self-reported BMI, physical activity, drinking, and smoking; and model 3 was a full model adding all trajectories. Moreover, considering that chronic diseases can impair both functional independence and cognitive performance, we further adjusted for various diseases, including cardiovascular diseases (stroke, hypertension, heart disease) and other diseases (type 2 diabetes, chronic lung disease, arthritis, psychological diseases, and cancer), to test the robustness of the results.

We conducted subgroup analysis based on age, gender, educational level, marital status, residence, total wealth income, self-reported BMI, physical activity, drinking, smoking, and all trajectories. In order to fully explore the combined effects of these variables, we selected significant risk trajectories and calculated their total scores. We then observed the impact of the total scores on the outcome. All analyses were done using R software, version 4.2.1 for Windows. Two-sided *P* values less than 0.05 were considered statistically significant.

## Results

### Trajectories of sleep disturbances, depressive symptoms, ADLs limitations, IADLs limitations and multimorbidity status

Of 4319 participants, the mean age was 75.47 years old (SD = 5.55) and 2653 (61.4%) were female. We identified two trajectories of sleep disturbances (Additional file 1: Fig. S1): one characterized by maintaining low sleep disturbances score (low; 3194 [74%]) and the other characterized by maintaining high scores throughout the follow-up period (high; 1125 [26%]). Compared with individuals in the low trajectory, those in the high trajectory were more likely to be older, female, and less educated and to engage in no physical activity, not drink, not smoke, have an increasing trajectory of depressive symptoms and IADLs limitations, and have a high trajectory of multimorbidity status (Additional file 1: Table S1).

**Fig. 2:**
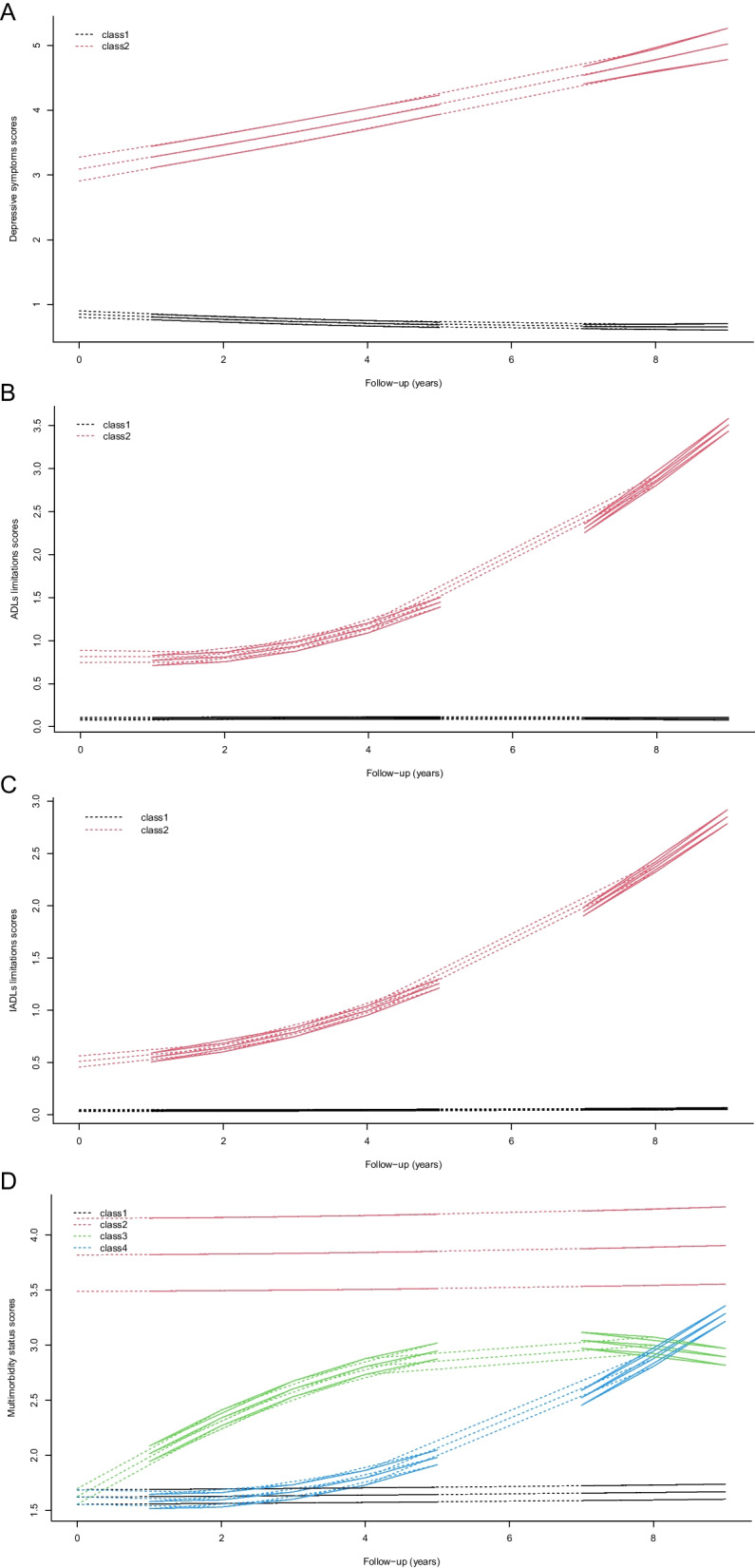
Trajectories of depressive symptoms, ADLs limitations, IADLs limitations, and multimorbidity status from 2002–2010. **A** Depressive symptoms: class 1 (black line, maintaining low CES-D score) and class 2 (red line, low starting CES-D scores that steadily increased throughout follow-up). **B** ADLs limitations: class 1 (black line, maintaining low ADLs score) and class 2 (red line, low starting ADLs scores that steadily increased throughout follow-up). **C** IADLs limitations: class 1 (black line, maintaining low IADLs score) and class 2 (red line, low starting IADLs scores that steadily increased throughout follow-up). **D** Multimorbidity status: class 1 (black line, maintaining low multimorbidity status), class 2 (red line, maintaining high multimorbidity status throughout follow-up), class 3 (green line, low starting multimorbidity status that increased early), and class 4 (blue line, low starting multimorbidity status that increased late). ADLs, activities of daily living; CES-D, Centers for Epidemiologic Research Depression; IADLs, instrumental activities of daily living

The trajectories of depressive symptoms were characterized by two patterns. The majority of participants maintained low CES-D scores (low; 3906 [90.4%]), and a smaller percentage had low starting scores that steadily increased throughout follow-up (increasing; 413 [9.6%]) (Fig. 2A). Compared with individuals in the low trajectories, those in the increasing trajectory were more likely to be female, less educated, and unmarried and to have lower income and abnormal BMI, engage in no physical activity, not drink, smoke, have a high trajectory of sleep disturbances and multimorbidity status, and have an increasing trajectory of ADLs limitations and IADLs limitations (Additional file 1: Table S2).

Both the trajectories of ADLs limitations and IADLs limitations were characterized by maintaining low scores (ADLs limitations = 4080 [94.5%]; IADLs limitations = 3906 [90.4%]) and initial low scores that steadily increased throughout the follow-up period (ADLs limitations = 239 [5.5%]; IADLs limitations = 3906 [90.4%]) (Fig. 2B, C). Compared with individuals in the low trajectories, those in the increasing trajectory of ADLs limitations and IADLs limitations were both more likely to be older, female, less educated, and unmarried and to have lower income, engage in no physical activity, and have no history of drinking or smoking (Additional file 1: Table S3 and Table S4). Individuals with an increasing trajectory of ADLs limitations were also more likely to exhibit a high trajectory of sleep disturbances and multimorbidity status, as well as an increasing trajectory of depressive symptoms and IADLs limitations. Meanwhile, individuals with an increasing trajectory of IADLs limitations were more likely to exhibit an increasing trajectory of depressive symptoms and ADLs limitations, as well as a high trajectory of multimorbidity status (Additional file 1: Table S3 and Table S4).

The trajectories of multimorbidity status were characterized by maintaining low multimorbidity status (low; 2073 [48.0%]), low starting scores that increased early (increasing early; 961 [22.3%]), low starting scores that increased late (increasing late; 1106 [25.6%]), and maintaining high scores throughout follow-up (high; 179 [4.1%]) (Fig. 2D). Compared with individuals in the other trajectories, those in the high trajectory were more likely to be female, to have lower education levels and lower income, to experience obesity, to engage in no physical activity, to not drink, smoke, to have a high trajectory of sleep disturbances, and to have an increasing trajectory of depressive symptoms, ADLs limitations, and IADLs limitations (Additional file 1: Table S5).

### The associations of trajectories of sleep disturbances, depressive symptoms, ADLs limitations, IADLs limitations and multimorbidity status with the risk of cognitive impairment

During 25,914 person-years, 1230 participants developed cognitive impairment. Older adults with cognitive impairment were more likely to be older, female, less educated, and unmarried and to live in rural areas, have less income, report no physical activity and drinking, and smoke (Table 1).

**Table 1:** Comparison of baseline characteristics

Groups	Overall (n, %)	Normal cognitive function (n, %)	Cognitive impairment (n, %)	*P*
**N**	4319	3089 (71.5)	1230 (28.5)	
**Age group (years)**				< 0.001
< 70	548 (12.7)	480 (15.5)	68 (5.5)	
≥ 80	970 (22.5)	532 (17.2)	438 (35.6)	
70 ~ 79	2801 (64.9)	2077 (67.2)	724 (58.9)	
**Gender**				0.582
Female	2653 (61.4)	1889 (61.2)	764 (62.1)	
Male	1666 (38.6)	1200 (38.8)	466 (37.9)	
**Educational level**				< 0.001
Less than high school	544 (12.6)	288 (9.3)	256 (20.8)	
High school or associate degree	1700 (39.4)	1179 (38.2)	521 (42.4)	
Some college or associate degree	995 (23.0)	755 (24.4)	240 (19.5)	
College degree or above	1080 (25.0)	867 (28.1)	213 (17.3)	
**Marital status**				< 0.001
Married	2516 (58.3)	1885 (61.0)	631 (51.3)	
Unmarried	1803 (41.7)	1204 (39.0)	599 (48.7)	
**Residence**				0.37
Rural	1349 (31.2)	952 (30.8)	397 (32.3)	
Urban	2970 (68.8)	2137 (69.2)	833 (67.7)	
**Total wealth income**				< 0.001
Lowest	451 (10.4)	288 (9.3)	163 (13.3)	
Quartile 2	916 (21.2)	625 (20.2)	291 (23.7)	
Quartile 3	1396 (32.3)	1007 (32.6)	389 (31.6)	
Highest	1556 (36.0)	1169 (37.8)	387 (31.5)	
**BMI (kg/m**^**2**^**)**				0.073
Underweight	50 (1.2)	36 (1.2)	14 (1.1)	
Normal	1266 (29.3)	881 (28.5)	385 (31.3)	
Obesity	968 (22.4)	678 (21.9)	290 (23.6)	
Overweight	2035 (47.1)	1494 (48.4)	541 (44.0)	
**Physical activity**				< 0.001
No	843 (19.5)	539 (17.4)	304 (24.7)	
Yes	3476 (80.5)	2550 (82.6)	926 (75.3)	
**Drinking**				< 0.001
No	1989 (46.1)	1315 (42.6)	674 (54.8)	
Yes	2330 (53.9)	1774 (57.4)	556 (45.2)	
**Smoking**				0.501
No	1982 (45.9)	1428 (46.2)	554 (45.0)	
Yes	2337 (54.1)	1661 (53.8)	676 (55.0)	

*BMI* Body mass index

In the univariate model, we found that compared with the low trajectory, the high trajectory of sleep disturbances (HR = 1.19; 95% CI = 1.05–1.35) and multimorbidity status (HR = 1.89; 95% CI = 1.49–2.39) were both associated with a higher risk of cognitive impairment (Table 2). Using the low trajectory as the reference trajectory, we found that individuals in the increasing trajectory of depressive symptoms (HR = 1.80; 95% CI = 1.53–2.12), ADLs limitations (HR = 1.88; 95% CI = 1.54–2.31), and IADLs limitations (HR = 2.98; 95% CI = 2.34–3.78) all had a higher risk of cognitive impairment (Table 2).

**Table 2:** Trajectories of sleep disturbances, depressive symptoms, ADLs limitations, IADLs limitations, and multimorbidity status and risk of cognitive impairment

	Cases/*N* (%)	Model 1	Model 2	Model 3
**Sleep disturbances**				
Low	877/3194 (27.5)	1 (Ref.)	1 (Ref.)	1 (Ref.)
High	353/1125 (31.4)	1.19 (1.05, 1.35)	1.05 (0.92, 1.19)	1.01 (0.89, 1.15)
**Depressive symptoms**				
Low	1062/3906 (27.2)	1 (Ref.)	1 (Ref.)	1 (Ref.)
Increasing	168/413 (40.7)	1.80 (1.53, 2.12)	1.46 (1.23, 1.73)	1.39 (1.17, 1.65)
**Multimorbidity status**				
Low	569/2073 (27.4)	1 (Ref.)	1 (Ref.)	1 (Ref.)
Increasing early	270/961 (28.1)	1.04 (0.90, 1.20)	0.95 (0.82, 1.09)	0.95 (0.82, 1.10)
Increasing late	313/1106 (28.3)	1.06 (0.92, 1.21)	0.94 (0.82, 1.08)	0.92 (0.80, 1.06)
High	78/179 (43.6)	1.89 (1.49, 2.39)	1.51 (1.19, 1.92)	1.48 (1.16, 1.88)
**ADLs limitations**				
Low	1128/4080 (27.6)	1 (Ref.)	1 (Ref.)	1 (Ref.)
Increasing	102/239 (42.7)	1.88 (1.54, 2.31)	1.40 (1.14, 1.73)	1.13 (0.88, 1.43)
**IADLs limitations**				
Low	1158/4193 (27.6)	1 (Ref.)	1 (Ref.)	1 (Ref.)
Increasing	72/126 (57.1)	2.98 (2.34, 3.78)	2.06 (1.61, 2.63)	1.88 (1.43, 2.46)

Model 1 was a univariate model; model 2 additionally adjusted for age, gender, educational level, marital status, residence, total wealth income, self-reported body mass index, physical activity, drinking, and smoking; and model 3 additionally added all trajectories. Data were represented as HR and 95%CI

*ADLs* Activities of daily living, *HR* Hazard ratio, *IADLs* Instrumental activities of daily living, *95%CI* 95% confidence interval

In the fully adjusted model 3, the trajectories of sleep disturbances and ADLs limitations were not associated with the risk of cognitive impairment. Compared to the low trajectory, we found that the high trajectory of multimorbidity status (HR = 1.48; 95% CI = 1.16–1.88), the increasing trajectory of depressive symptoms (HR = 1.39; 95% CI = 1.17–1.65), and the increasing trajectory of IADLs limitations (HR = 1.88; 95% CI = 1.43–2.46) still presented a higher risk of cognitive impairment (Table 2). After adjusting for different diseases, such as cardiovascular diseases and others, the results showed that the association between the trajectories of sleep disturbances, depressive symptoms, ADLs limitations, and IADLs limitations, and the risk of cognitive impairment remained stable (Additional file 1: Table S6).

**Fig. 3:**
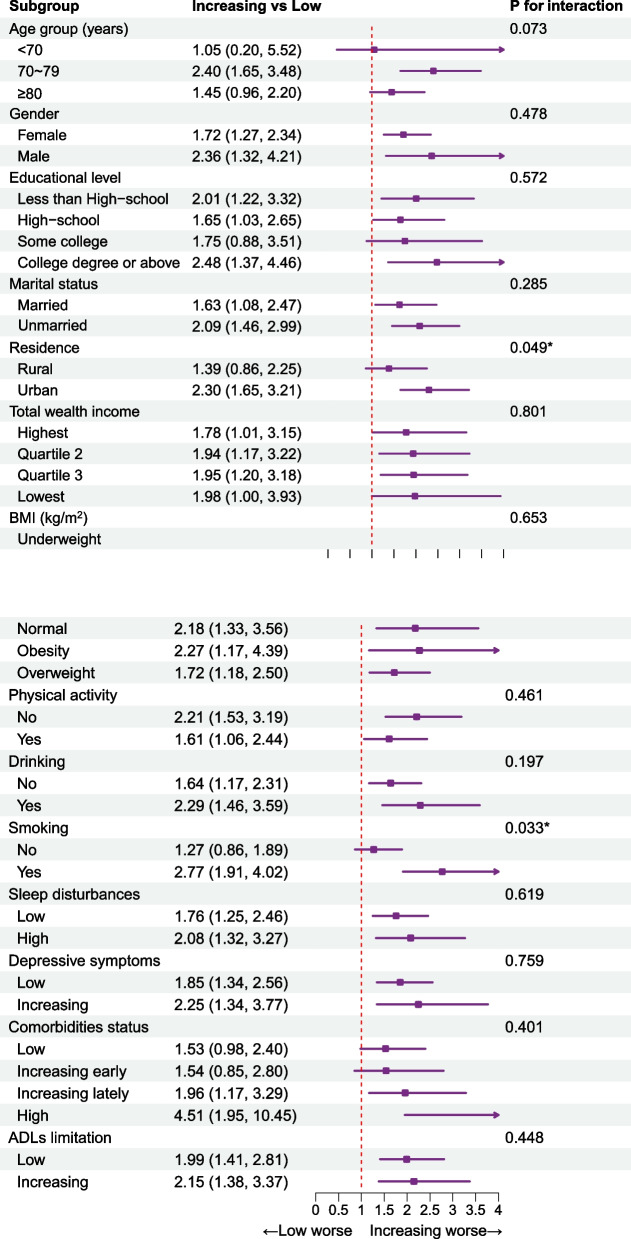
Subgroup analysis of associations between the trajectories of IADLs limitations and risk of cognitive impairment. Notes: all models were model 3. Data were represented as HR and 95%CI. *BMI*, body mass index; *HR*, hazard ratio; *IADLs*, instrumental activities of daily living; *95%CI*, 95% confidence interval

Subgroup analysis showed that there were no statistically significant differences among subgroups in terms of the trajectories of sleep disturbances, depressive symptoms, multimorbidity status, and ADLs limitations (Additional file 1: Table S7- Table S10), except the trajectories of IADLs limitations (Fig. 3). Compared with older populations who lived in rural areas (HR = 1.39; 95% CI = 0.86–2.25), the increasing trajectory of IADLs limitations posed a higher risk of cognitive impairment for those who lived in urban areas (HR = 2.30; 95% CI = 1.65–3.21; *P* for interaction = 0.049). Compared with older adults who never smoked (HR = 1.27; 95% CI = 0.86–1.89), the increasing trajectory of IADLs limitations posed a higher risk of cognitive impairment for those who smoked (HR = 2.77; 95% CI = 1.91–4.02;* P* for interaction = 0.033).

Based on the above results, we calculated the total scores of trajectories, including depressive symptoms (low = 0; high = 1), multimorbidity status (low, increasing early, increasing late = 0; high = 1), and IADLs limitations (low = 0; increasing = 1). The total scores of risk trajectories ranged from 0 to 3. For every point increase in the risk trajectories, the risk of cognitive impairment increased by 49% (HR = 1.49; 95% CI = 1.32–1.67).

## Discussion

In our 16-year cohort study, we identified two trajectories of sleep disturbances (low, high), depressive symptoms (low, increasing), ADLs limitations (low, increasing), and IADLs limitations (low, increasing), as well as four trajectories of multimorbidity status (low, increasing early, increasing late, high). The high trajectory of multimorbidity status, increasing trajectory of depressive symptoms, and increasing trajectory of IADLs limitations all posed a higher risk of cognitive impairment. Furthermore, the increasing trajectory of IADLs limitations had a higher risk of cognitive impairment for older populations who lived in urban areas and smoked. For every point increase in the total scores of risk trajectories, which included multimorbidity status, depressive symptoms, and IADLs limitations, the risk of cognitive impairment increased by 49%.

The varying trajectories of depressive symptoms, multimorbidity status, and IADLs limitations over nearly 8 years predicted the risks of cognitive impairment. We observed that individuals with increasing depressive symptoms had a significantly higher risk of cognitive impairment. Mirza et al. reported that only increasing depressive symptoms was associated with a higher risk of dementia, compared to the low depressive symptom trajectory, which supported our findings [12]. Mirza et al. proposed that depressive symptoms might lie in a continuum between subclinical cognitive impairment and overt dementia [12, 26, 27]. Kong et al. reported that older adults with increasing and persistently high depressive symptoms may experience worse memory [28]. By identifying patterns in the progression of depressive symptoms in our study, we found that the gradual escalation of depressive symptoms started several years before cognitive impairment occurred. But the important thing to note is that there is often a bidirectional relationship between depression and cognitive impairment, meaning that cognitive impairment in its early stages often leads to the onset of depression. The specific mechanisms of the impact of depressive symptoms on cognitive impairment are relatively few and complex. Inflammatory, trophic, and cerebrovascular factors may contribute, in addition to monoamine deficiency and the severity of plaques and tangle pathology [29].

Furthermore, we found that increasing IADL limitations, rather than ADL limitations increased the risk of cognitive impairment. Similarly, Sun et al. reported that the loss of ADL limitations among older adults was not found to be a significant risk factor for cognitive function [30]. Meanwhile, a study utilizing a group-based trajectory model indicated that the high-risk IADL group increased the risk of mild cognitive impairment among Chinese older individuals [14]. However, a bidirectional relationship might exist between cognitive function and functional limitations. Zang et al. reported that persons with dementia experienced steady declines in physical functioning [31]. In our study, we assessed IADL limitations using five instrumental activities of daily living, including using a telephone, managing money, taking medications, shopping for groceries, and preparing hot meals. Our findings reported an association between IADL limitations and cognitive impairment, but not ADL limitations. This might indicate that IADL limitations were the pre-cognitive impairment performances, similar to depressive symptoms [31]. Besides, it may be economical and convenient to adopt the IADL limitations as a supplementary screening tool for high-risk populations who develop cognitive impairment. However, we still need to explore the bidirectional relationship between physical functioning and cognitive impairment using appropriate causal inference methods in the future. We found that high multimorbidity status was a risk factor for cognitive impairment. Cross-sectional studies reported that multimorbidity was correlated with cognitive impairment [32, 33]. Limited studies have explored the association between comorbidity at multiple time points and cognitive impairment among older adults. Jones et al. reported that the severity of multimorbidity including arthritis, heart/circulation problems, and diabetes contributed to poorer delayed recall performance and lower semantic fluency scores in a cohort study [34]. Chronic co-morbidities, such as arthritis and diabetes, are risk factors for subsequent dementia due to their poorer progression of high inflammatory status [35].

After adjusting for covariates, it was found that high sleep disturbance and increasing ADLs limitations were not associated with the risk of cognitive impairment. However, the incidence of cognitive impairment was higher among older populations experiencing high sleep disturbances and increasing ADLs limitations, compared with those who reported low sleep disturbances and ADLs limitations, respectively. Zitser et al. also found that there was no significant association between sleep duration patterns and cognitive function or brain structure [36]. Previous studies have found that sleep disturbances and ADLs limitations were associated with risk of cognitive impairment when measuring them at a single point [7–9]. Our findings may indicate that more research is necessary to comprehend the impact of sleep disturbances and ADLs limitations on the risk of cognitive impairment. It is important to explore possible influencing factors within longitudinal data, rather than measuring them at a single point. For example, Guo et al. found that a persistent short nighttime sleep duration trajectory and persistent seldom daytime napping duration were associated with a higher risk of multimorbidity [37]. Multimorbidity may mediate the associations between sleep disturbances and cognitive impairment when the study measures sleep only once.

Compared with individuals in the low trajectories, those in the increasing trajectory of depressive symptoms and IADLs limitations were more likely to be female, less educated, and unmarried and to have lower income and engage in less physical activity. Meanwhile, compared with individuals in the other trajectories, those in the high trajectory of multimorbidity status were also more likely to be female and less educated, to have lower income, and to engage in less physical activity. Previous studies have also reported similar findings [12, 23]. Our results indicate that female, older adults with lower educational level and income should be the key population at risk for experiencing the poor course of depressive symptoms, functional limitations, and multimorbidity status. Besides, the older population may experience a decrease in their ability to engage in physical activity due to exacerbated depressive symptoms, functional limitations, and multimorbidity status. Consequently, older adults at risk trajectories engaged in less physical activity.

Population aging is associated with the co-occurrence of physical and psychological disorders [5]. In general, early screening and detection of cognitive impairment is more cost-effective and convenient than the clinical diagnosis of dementia. Identification of risk factors is essential for timely intervention to delay cognitive decline. Our study suggests that the trajectory of depressive symptoms, IADLs limitations, and multimorbidity may be risk factors for cognitive impairment. This strategy can be useful for more accurately identifying individuals at risk for cognitive impairment than a single time-point observation, as we know that exposure to risk factors can vary over time. In order to reduce the risk of cognitive impairment, observing the daily activities and psychological status of the elderly, as well as recording the multimorbidity, is conducive to providing critical interventions for high-risk populations.

### Strengths and limitations

The major strength of this study was its ability to identify different courses of sleep disturbances, depressive symptoms, ADLs limitations, IADLs limitations, and multimorbidity status, and their relationship to the development of cognitive impairment. However, this study still had several limitations. Firstly, we used self-reported data on cognitive impairment and multimorbidity. Although multimorbidity was self-reported clinical diagnosis, the absence of a diagnosis record may introduce recall bias. Secondly, we only considered other covariates, such as physical activity, drinking, and smoking at a single point. However, these factors could change over time as potential risk factors for cognitive impairment. Furthermore, some relevant characteristics, such as social factors, were not measured in our study. Further research on the relationship between the observed trajectories and cognitive impairment would benefit from investigating these characteristics, including the changes in lifestyle habits and social factors. Exploring the impact of these factors on the association between health status and the development of cognitive impairment using cohort studies, randomized controlled trials (RCT), or structural equation analysis is crucial for improving cognitive impairment in the future. Finally, it should be noted that dementia is defined by the presence of cognitive decline sufficient enough to interfere with independence. It is possible that functional decline is not a risk factor but rather a manifestation of the onset of cognitive impairment. We conducted a cohort study to present that the decline in physical function may increase the risk of cognitive impairment after controlling other trajectories. In addition, the ADLs limitations and IADLs limitations primarily assess the activities of daily living, while the cognitive scale primarily assesses memory, which does not fully represent a clinical diagnosis of dementia. On the contrary, the use of this cognitive scale in a large-sample cohort study may reduce this possibility. In order to clarify the bidirectional relationship between physical disorders and mental health in the future, RCT is needed to explore the effect of early intervention for functional impairment on cognitive impairment.

## Conclusions

In conclusion, the increased risk of subsequent cognitive impairment among individuals with exasperate courses of depressive symptoms, instrumental functioning limitations, and multimorbidity suggests that we should pay more attention to the impact of long-term changes in health status on mental disorders. The courses of depressive symptoms, instrumental functioning limitations, and multimorbidity may serve as effective screening and warning indicators of cognitive impairment in the daily care of older adults. Future studies are warranted to unravel the biological underpinnings of these associations. In addition, causal inference methods could be used to investigate the potential of using trajectories (as opposed to single assessments) as a screening method to identify older adults at risk of cognitive impairment and intervene early.

### Supplementary Information

**Additional file 1: Fig. S1.** Trajectories of sleep disturbances. **Table S1.** The participants’ characteristics stratified by trajectories of sleep disturbances. **Table S2.** The participants’ characteristics stratified by trajectories of depressive symptoms. **Table S3.** The participants’ characteristics stratified by trajectories of ADLs limitations. **Table S4.** The participants’ characteristics stratified by trajectories of IADLs limitations. **Table S5.** The participants’ characteristics stratified by trajectories of multimorbidity status. **Table S6.** Trajectories of sleep disturbances, depressive symptoms, ADLs limitations, and IADLs limitations and risk of cognitive impairment. **Table S7.** Subgroup analysis of the trajectories of sleep disturbances and risk of cognitive impairment. **Table S8.** Subgroup analysis of the trajectories of depressive symptoms and risk of cognitive impairment. **Table S9.** Subgroup analysis of the trajectories of multimorbidity status and risk of cognitive impairment. **Table S10.** Subgroup analysis of the trajectories of ADLs limitations and risk of cognitive impairment.

## Data Availability

The HRS datasets are openly available from https://hrsdata.isr.umich.edu/data-products/public-survey-data (accessed on 10 May 2023).

## Abbreviations

ADLs: Activities of daily living
BIC: Bayesian Information Criterion
BMI: Body mass index
CES-D: Center for Epidemiology Depression Scale
CI: Confidence interval
HR: Hazard ratios
HRS: Health and Retirement Study
IADLs: Instrumental activities of daily living
LCTM: Latent class trajectory models
RCT: Randomized controlled trials
SD: Standard deviation
