# Supplementary material for: Trajectories of health conditions and their associations with the risk of cognitive impairment among older adults: insights from a national prospective cohort study
Source: BMC Med. 2024 Jan 10;22:20. doi: 10.1186/s12916-024-03245-x (PMC10777570; doi:10.1186/s12916-024-03245-x)
Supplement: Supplementary file 1 — Additional file 1: Fig. S1. Trajectories of sleep disturbances. Table S1. The participants’ characteristics stratified by trajectories of sleep disturbances. Table S2. The participants’ characteristics stratified by trajectories of depressive symptoms. Table S3. The participants’ characteristics stratified by trajectories of ADLs limitations. Table S4. The participants’ characteristics stratified by trajectories of IADLs limitations. Table S5. The participants’ characteristics stratified by trajectories of multimorbidity status. Table S6. Trajectories of sleep disturbances, depressive symptoms, ADLs limitations, and IADLs limitations and risk of cognitive impairment. Table S7. Subgroup analysis of the trajectories of sleep disturbances and risk of cognitive impairment. Table S8. Subgroup analysis of the trajectories of depressive symptoms and risk of cognitive impairment. Table S9. Subgroup analysis of the trajectories of multimorbidity status and risk of cognitive impairment. Table S10. Subgroup analysis of the trajectories of ADLs limitations and risk of cognitive impairment. [file 12916_2024_3245_MOESM1_ESM.docx]

**Additional File 1**

[**Fig.S1**. Trajectories of sleep disturbances 2](#_Toc152844561)

[**Table** **S1**. The participants’ characteristics stratified by trajectories of sleep disturbances 3](#_Toc152844562)

[**Table** **S2**. The participants’ characteristics stratified by trajectories of depressive symptoms 5](#_Toc152844563)

[**Table** **S3**. The participants’ characteristics stratified by trajectories of ADLs limitations 7](#_Toc152844564)

[**Table S4**. The participants’ characteristics stratified by trajectories of IADLs limitations 9](#_Toc152844565)

[**Table** **S5**. The participants’ characteristics stratified by trajectories of multimorbidity status 11](#_Toc152844566)

[**Table** **S6**. Trajectories of sleep disturbances, depressive symptoms, ADLs limitations, and IADLs limitations and risk of cognitive impairment 13](#_Toc152844567)

[**Table** **S7**. Subgroup analysis of the trajectories of sleep disturbances and risk of cognitive impairment 14](#_Toc152844568)

[**Table** **S8**. Subgroup analysis of the trajectories of depressive symptoms and risk of cognitive impairment 16](#_Toc152844569)

[**Table** **S9**. Subgroup analysis of the trajectories of multimorbidity status and risk of cognitive impairment 18](#_Toc152844570)

[**Table** **S10**. Subgroup analysis of the trajectories of ADLs limitations and risk of cognitive impairment 20](#_Toc152844571)


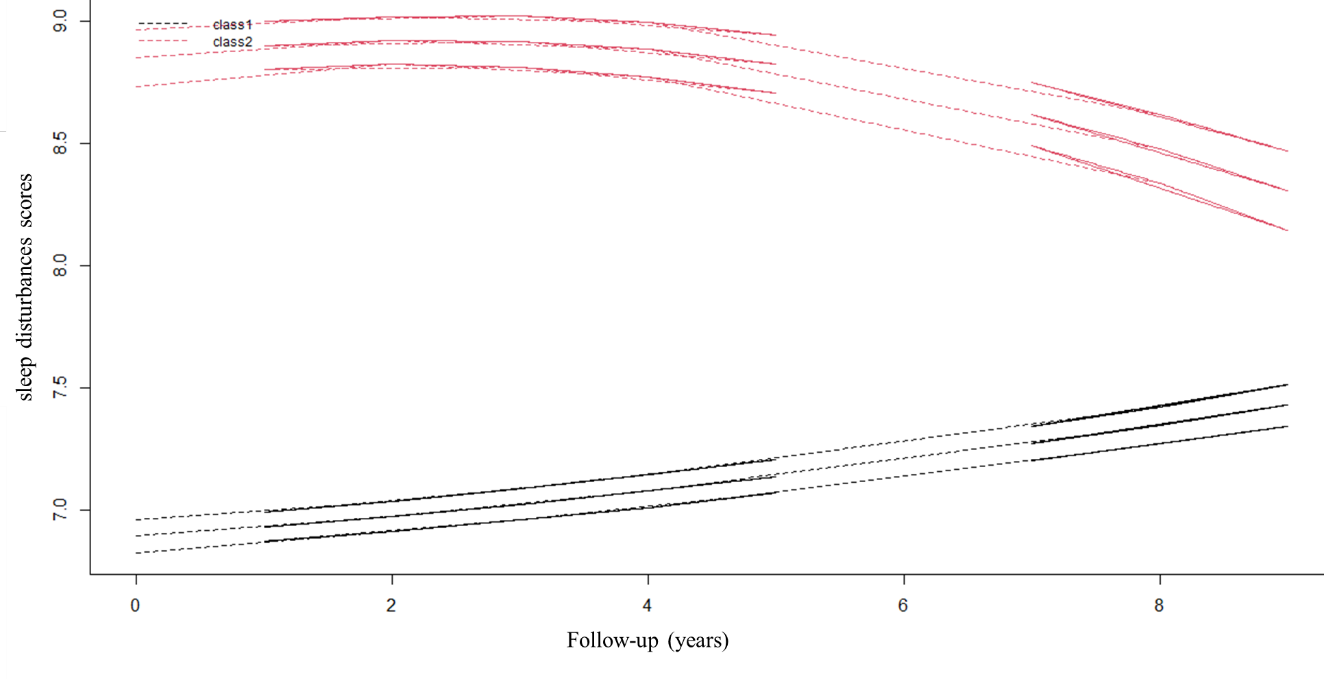


**Fig.S1. Trajectories of sleep disturbances**

Notes: class 1(black line, maintaining low sleep disturbances score) and class 2 (red line, maintaining high sleep disturbances scores throughout follow up)

**Table S1. The** **participants’ characteristics stratified by trajectories of sleep disturbances**

| **Groups** | **Overall** | **Low** | **High** | **P** |
| --- | --- | --- | --- | --- |
| **Sleep disturbances** | 4319 | 3194 (74.0) | 1125 (26.0) | |
| **Age group (years)** | |  |  | 0.001 |
| <70 | 548 (12.7) | 426 (13.3) | 122 (10.8) |  |
| 70~79 | 2801 (64.9) | 2091 (65.5) | 710 (63.1) |  |
| ≥80 | 970 (22.5) | 677 (21.2) | 293 (26.0) |  |
| **Gender** |  |  |  | <0.001 |
| Female | 2653 (61.4) | 1830 (57.3) | 823 (73.2) |  |
| Male | 1666 (38.6) | 1364 (42.7) | 302 (26.8) |  |
| **Educational level** | |  |  | <0.001 |
| Some college or associate degree | 995 (23.0) | 738 (23.1) | 257 (22.8) |  |
| College degree or above | 1080 (25.0) | 868 (27.2) | 212 (18.8) |  |
| High-school or associate degree | 1700 (39.4) | 1215 (38.0) | 485 (43.1) |  |
| Less than High-school | 544 (12.6) | 373 (11.7) | 171 (15.2) |  |
| **Marital status** | |  |  | <0.001 |
| Married | 2516 (58.3) | 1933 (60.5) | 583 (51.8) |  |
| Unmarried | 1803 (41.7) | 1261 (39.5) | 542 (48.2) |  |
| **Residence** | |  |  | 0.084 |
| Rural | 1349 (31.2) | 974 (30.5) | 375 (33.3) |  |
| Urban | 2970 (68.8) | 2220 (69.5) | 750 (66.7) |  |
| **Total wealth income** | | |  | 0.806 |
| Highest | 1556 (36.0) | 1141 (35.7) | 415 (36.9) |  |
| Quartile 3 | 1396 (32.3) | 1045 (32.7) | 351 (31.2) |  |
| Quartile 2 | 916 (21.2) | 677 (21.2) | 239 (21.2) |  |
| Lowest | 451 (10.4) | 331 (10.4) | 120 (10.7) |  |
| **BMI (kg/m^2^)** | |  |  | 0.091 |
| Underweight | 50 (1.2) | 34 (1.1) | 16 (1.4) |  |
| Normal | 1266 (29.3) | 912 (28.6) | 354 (31.5) |  |
| Obesity | 968 (22.4) | 740 (23.2) | 228 (20.3) |  |
| Overweight | 2035 (47.1) | 1508 (47.2) | 527 (46.8) |  |
| **Physical activity** | |  |  | <0.001 |
| No | 843 (19.5) | 576 (18.0) | 267 (23.7) |  |
| Yes | 3476 (80.5) | 2618 (82.0) | 858 (76.3) |  |
| **Drinking** |  |  |  | 0.008 |
| No | 1989 (46.1) | 1432 (44.8) | 557 (49.5) |  |
| Yes | 2330 (53.9) | 1762 (55.2) | 568 (50.5) |  |
| **Smoking** |  |  |  | 0.106 |
| No | 1982 (45.9) | 1442 (45.1) | 540 (48.0) |  |
| Yes | 2337 (54.1) | 1752 (54.9) | 585 (52.0) |  |
| **Depressive symptoms** |  |  |  | <0.001 |
| Low | 3906 (90.4) | 2952 (92.4) | 954 (84.8) |  |
| Increasing | 413 (9.6) | 242 (7.6) | 171 (15.2) |  |
| **Multimorbidity status** | |  |  | <0.001 |
| Low | 2073 (48.0) | 1567 (49.1) | 506 (45.0) |  |
| Increasing early | 961 (22.3) | 693 (21.7) | 268 (23.8) |  |
| Increasing late | 1106 (25.6) | 824 (25.8) | 282 (25.1) |  |
| High | 179 (4.1) | 110 (3.4) | 69 (6.1) |  |
| **ADLs limitations** | |  |  | <0.001 |
| Low | 4080 (94.5) | 3048 (95.4) | 1032 (91.7) |  |
| Increasing | 239 (5.5) | 146 (4.6) | 93 (8.3) |  |
| **IADLs limitations** | |  |  | 0.169 |
| Low | 4193 (97.1) | 3108 (97.3) | 1085 (96.4) |  |
| Increasing | 126 (2.9) | 86 (2.7) | 40 (3.6) |  |

Notes: ADLs =activities of daily living. BMI = body mass index. IADLs=instrumental activities of daily living.

**Table S2. The participants’ characteristics stratified by trajectories of depressive symptoms**

| **Groups** | **Overall** | **Low** | **Increasing** | **P** |
| --- | --- | --- | --- | --- |
| **Depressive symptoms** | 4319 | 3906 (90.4) | 413 (9.6) |  |
| **Age group (years)** | |  |  | 0.988 |
| <70 | 548 (12.7) | 496 (12.7) | 52 (12.6) |  |
| 70~79 | 2801 (64.9) | 2534 (64.9) | 267 (64.6) |  |
| ≥80 | 970 (22.5) | 876 (22.4) | 94 (22.8) |  |
| **Gender** |  |  |  | <0.001 |
| Female | 2653 (61.4) | 2334 (59.8) | 319 (77.2) |  |
| Male | 1666 (38.6) | 1572 (40.2) | 94 (22.8) |  |
| **Educational level** | |  |  | <0.001 |
| College degree or above | 1080 (25.0) | 1021 (26.1) | 59 (14.3) |  |
| Some college or associate degree | 995 (23.0) | 898 (23.0) | 97 (23.5) |  |
| High-school or associate degree | 1700 (39.4) | 1534 (39.3) | 166 (40.2) |  |
| Less than High-school | 544 (12.6) | 453 (11.6) | 91 (22.0) |  |
| **Marital status** | |  |  | <0.001 |
| Married | 2516 (58.3) | 2369 (60.7) | 147 (35.6) |  |
| Unmarried | 1803 (41.7) | 1537 (39.3) | 266 (64.4) |  |
| **Residence** | |  |  | 0.132 |
| Rural | 1349 (31.2) | 1234 (31.6) | 115 (27.8) |  |
| Urban | 2970 (68.8) | 2672 (68.4) | 298 (72.2) |  |
| **Total wealth income** | | |  | <0.001 |
| Highest | 1556 (36.0) | 1454 (37.2) | 102 (24.7) |  |
| Quartile 2 | 916 (21.2) | 793 (20.3) | 123 (29.8) |  |
| Quartile 3 | 1396 (32.3) | 1284 (32.9) | 112 (27.1) |  |
| Lowest | 451 (10.4) | 375 (9.6) | 76 (18.4) |  |
| **BMI (kg/m^2^)** | |  |  | 0.03 |
| Underweight | 50 (1.2) | 44 (1.1) | 6 (1.5) |  |
| Normal | 1266 (29.3) | 1155 (29.6) | 111 (26.9) |  |
| Obesity | 968 (22.4) | 893 (22.9) | 75 (18.2) |  |
| Overweight | 2035 (47.1) | 1814 (46.4) | 221 (53.5) |  |
| **Physical activity** | |  |  | <0.001 |
| No | 843 (19.5) | 694 (17.8) | 149 (36.1) |  |
| Yes | 3476 (80.5) | 3212 (82.2) | 264 (63.9) |  |
| **Drinking** |  |  |  | <0.001 |
| No | 1989 (46.1) | 1754 (44.9) | 235 (56.9) |  |
| Yes | 2330 (53.9) | 2152 (55.1) | 178 (43.1) |  |
| **Smoking** |  |  |  | 0.119 |
| No | 1982 (45.9) | 1808 (46.3) | 174 (42.1) |  |
| Yes | 2337 (54.1) | 2098 (53.7) | 239 (57.9) |  |
| **Sleep disturbances** |  |  |  | <0.001 |
| Low | 3194 (74.0) | 2952 (75.6) | 242 (58.6) |  |
| High | 1125 (26.0) | 954 (24.4) | 171 (41.4) |  |
| **Multimorbidity status** | |  |  | <0.001 |
| Low | 2073 (48.0) | 1927 (49.3) | 146 (35.4) |  |
| Increasing early | 961 (22.3) | 849 (21.7) | 112 (27.1) |  |
| Increasing late | 1106 (25.6) | 984 (25.2) | 122 (29.5) |  |
| High | 179 (4.1) | 146 (3.7) | 33 (8.0) |  |
| **ADLs limitations** | |  |  | <0.001 |
| Low | 4080 (94.5) | 3739 (95.7) | 341 (82.6) |  |
| Increasing | 239 (5.5) | 167 (4.3) | 72 (17.4) |  |
| **IADLs limitations** | |  |  | <0.001 |
| Low | 4193 (97.1) | 3823 (97.9) | 370 (89.6) |  |
| Increasing | 126 (2.9) | 83 (2.1) | 43 (10.4) |  |

Notes: ADLs =activities of daily living. BMI = body mass index. IADLs=instrumental activities of daily living.

**Table S3. The participants’ characteristics stratified by trajectories of ADLs limitations**

| **Groups** | **Overall** | **Low** | **Increasing** | **P** |
| --- | --- | --- | --- | --- |
| **ADLs limitations** | 4319 | 4080 (94.5) | 239 (5.5) |  |
| **Age group (years)** | |  |  | 0.004 |
| <70 | 548 (12.7) | 524 (12.8) | 24 (10.0) |  |
| 70~79 | 2801 (64.9) | 2660 (65.2) | 141 (59.0) |  |
| ≥80 | 970 (22.5) | 896 (22.0) | 74 (31.0) |  |
| **Gender** |  |  |  | <0.001 |
| Female | 2653 (61.4) | 2479 (60.8) | 174 (72.8) |  |
| Male | 1666 (38.6) | 1601 (39.2) | 65 (27.2) |  |
| **Educational level** | |  |  | <0.001 |
| College degree or above | 1080 (25.0) | 1038 (25.4) | 42 (17.6) |  |
| Some college or associate degree | 995 (23.0) | 941 (23.1) | 54 (22.6) |  |
| High-school or associate degree | 1700 (39.4) | 1607 (39.4) | 93 (38.9) |  |
| Less than High-school | 544 (12.6) | 494 (12.1) | 50 (20.9) |  |
| **Marital status** | |  |  | <0.001 |
| Married | 2516 (58.3) | 2413 (59.1) | 103 (43.1) |  |
| Unmarried | 1803 (41.7) | 1667 (40.9) | 136 (56.9) |  |
| **Residence** | |  |  |  |
| Rural | 1349 (31.2) | 1287 (31.5) | 62 (25.9) | 0.081 |
| Urban | 2970 (68.8) | 2793 (68.5) | 177 (74.1) |  |
| **Total wealth income** | | |  | <0.001 |
| Highest | 1556 (36.0) | 1487 (36.4) | 69 (28.9) |  |
| Quartile 2 | 916 (21.2) | 865 (21.2) | 51 (21.3) |  |
| Quartile 3 | 1396 (32.3) | 1338 (32.8) | 58 (24.3) |  |
| Lowest | 451 (10.4) | 390 (9.6) | 61 (25.5) |  |
| **BMI (kg/m^2^)** | |  |  | <0.001 |
| Underweight | 50 (1.2) | 49 (1.2) | 1 (0.4) |  |
| Normal | 1266 (29.3) | 1220 (29.9) | 46 (19.2) |  |
| Obesity | 968 (22.4) | 931 (22.8) | 37 (15.5) |  |
| Overweight | 2035 (47.1) | 1880 (46.1) | 155 (64.9) |  |
| **Physical activity** | |  |  | <0.001 |
| No | 843 (19.5) | 718 (17.6) | 125 (52.3) |  |
| Yes | 3476 (80.5) | 3362 (82.4) | 114 (47.7) |  |
| **Drinking** |  |  |  | 0.001 |
| No | 1989 (46.1) | 1854 (45.4) | 135 (56.5) |  |
| Yes | 2330 (53.9) | 2226 (54.6) | 104 (43.5) |  |
| **Smoking** |  |  |  | 0.913 |
| No | 1982 (45.9) | 1871 (45.9) | 111 (46.4) |  |
| Yes | 2337 (54.1) | 2209 (54.1) | 128 (53.6) |  |
| **Sleep disturbances** |  |  |  | <0.001 |
| Low | 3194 (74.0) | 3048 (74.7) | 146 (61.1) |  |
| High | 1125 (26.0) | 1032 (25.3) | 93 (38.9) |  |
| **Depressive symptoms** |  |  |  | <0.001 |
| Low | 3906 (90.4) | 3739 (91.6) | 167 (69.9) |  |
| Increasing | 413 (9.6) | 341 (8.4) | 72 (30.1) |  |
| **Multimorbidity status** | |  |  | <0.001 |
| Low | 2073 (48.0) | 1992 (48.8) | 81 (33.9) |  |
| Increasing early | 961 (22.3) | 897 (22.0) | 64 (26.8) |  |
| Increasing late | 1106 (25.6) | 1031 (25.3) | 75 (31.4) |  |
| High | 179 (4.1) | 160 (3.9) | 19 (7.9) |  |
| **IADLs limitations** | |  |  | <0.001 |
| Low | 4193 (97.1) | 4016 (98.4) | 177 (74.1) |  |
| Increasing | 126 (2.9) | 64 (1.6) | 62 (25.9) |  |

Notes: ADLs =activities of daily living. BMI = body mass index. IADLs=instrumental activities of daily living.

**Table S4. The participants’ characteristics stratified by trajectories of IADLs limitations**

| **Groups** | **Overall** | **Low** | **Increasing** | **P** |
| --- | --- | --- | --- | --- |
| **ADLs limitations** | 4319 | 4193 (97.1) | 126 (2.9) |  |
| **Age group (years)** | |  |  | <0.001 |
| <70 | 548 (12.7) | 536 (12.8) | 12 (9.5) |  |
| 70~79 | 2801 (64.9) | 2735 (65.2) | 66 (52.4) |  |
| ≥80 | 970 (22.5) | 922 (22.0) | 48 (38.1) |  |
| **Gender** |  |  |  | <0.001 |
| Female | 2653 (61.4) | 2553 (60.9) | 100 (79.4) |  |
| Male | 1666 (38.6) | 1640 (39.1) | 26 (20.6) |  |
| **Educational level** | |  |  | <0.001 |
| College degree or above | 1080 (25.0) | 1058 (25.2) | 22 (17.5) |  |
| Some college or associate degree | 995 (23.0) | 976 (23.3) | 19 (15.1) |  |
| High-school or associate degree | 1700 (39.4) | 1650 (39.4) | 50 (39.7) |  |
| Less than High-school | 544 (12.6) | 509 (12.1) | 35 (27.8) |  |
| **Marital status** | |  |  | 0.046 |
| Married | 2516 (58.3) | 2454 (58.5) | 62 (49.2) |  |
| Unmarried | 1803 (41.7) | 1739 (41.5) | 64 (50.8) |  |
| **Residence** | |  |  | 0.231 |
| Rural | 1349 (31.2) | 1303 (31.1) | 46 (36.5) |  |
| Urban | 2970 (68.8) | 2890 (68.9) | 80 (63.5) |  |
| **Total wealth income** | | |  | <0.001 |
| Highest | 1556 (36.0) | 1525 (36.4) | 31 (24.6) |  |
| Quartile 2 | 916 (21.2) | 875 (20.9) | 41 (32.5) |  |
| Quartile 3 | 1396 (32.3) | 1365 (32.6) | 31 (24.6) |  |
| Lowest | 451 (10.4) | 428 (10.2) | 23 (18.3) |  |
| **BMI (kg/m^2^)** | |  |  | 0.12 |
| Underweight | 50 (1.2) | 49 (1.2) | 1 (0.8) |  |
| Normal | 1266 (29.3) | 1233 (29.4) | 33 (26.2) |  |
| Obesity | 968 (22.4) | 948 (22.6) | 20 (15.9) |  |
| Overweight | 2035 (47.1) | 1963 (46.8) | 72 (57.1) |  |
| **Physical activity** | |  |  | <0.001 |
| No | 843 (19.5) | 770 (18.4) | 73 (57.9) |  |
| Yes | 3476 (80.5) | 3423 (81.6) | 53 (42.1) |  |
| **Drinking** |  |  |  | <0.001 |
| No | 1989 (46.1) | 1906 (45.5) | 83 (65.9) |  |
| Yes | 2330 (53.9) | 2287 (54.5) | 43 (34.1) |  |
| **Smoking** |  |  |  | 0.627 |
| No | 1982 (45.9) | 1921 (45.8) | 61 (48.4) |  |
| Yes | 2337 (54.1) | 2272 (54.2) | 65 (51.6) |  |
| **Sleep disturbances** |  |  |  | 0.169 |
| Low | 3194 (74.0) | 3108 (74.1) | 86 (68.3) |  |
| High | 1125 (26.0) | 1085 (25.9) | 40 (31.7) |  |
| **Depressive symptoms** |  |  |  | <0.001 |
| Low | 3906 (90.4) | 3823 (91.2) | 83 (65.9) |  |
| Increasing | 413 (9.6) | 370 (8.8) | 43 (34.1) |  |
| **Multimorbidity status** | |  |  | <0.001 |
| Low | 2073 (48.0) | 2027 (48.3) | 46 (36.5) |  |
| Increasing early | 961 (22.3) | 935 (22.3) | 26 (20.6) |  |
| Increasing late | 1106 (25.6) | 1065 (25.4) | 41 (32.5) |  |
| High | 179 (4.1) | 166 (4.0) | 13 (10.3) |  |
| **ADLs limitations** | |  |  | <0.001 |
| Low | 4080 (94.5) | 4016 (95.8) | 64 (50.8) |  |
| Increasing | 239 (5.5) | 177 (4.2) | 62 (49.2) |  |

Notes: ADLs =activities of daily living. BMI = body mass index. IADLs=instrumental activities of daily living.

**Table S5. The participants’ characteristics stratified by trajectories of multimorbidity status**

| **Groups** | **Overall** | **Low** | **Increased early** | **Increased late** | **High** | **P** |
| --- | --- | --- | --- | --- | --- | --- |
| **Multimorbidity status** | 4319 | 2073 (48.0) | 961 (22.3) | 1106 (25.6) | 179 (4.1) |  |
| **Age group (years)** | |  |  |  |  | 0.877 |
| <70 | 548 (12.7) | 264 (12.7) | 120 (12.5) | 145 (13.1) | 19 (10.6) |  |
| 70~79 | 2801 (64.9) | 1357 (65.5) | 620 (64.5) | 710 (64.2) | 114 (63.7) |  |
| ≥80 | 970 (22.5) | 452 (21.8) | 221 (23.0) | 251 (22.7) | 46 (25.7) |  |
| **Gender** |  |  |  |  |  | 0.035 |
| Female | 2653 (61.4) | 1303 (62.9) | 597 (62.1) | 639 (57.8) | 114 (63.7) |  |
| Male | 1666 (38.6) | 770 (37.1) | 364 (37.9) | 467 (42.2) | 65 (36.3) |  |
| **Educational level** | |  |  |  |  | 0.009 |
| College degree or above | 1080 (25.0) | 552 (26.6) | 231 (24.0) | 261 (23.6) | 36 (20.1) |  |
| Some college or associate degree | 995 (23.0) | 502 (24.2) | 215 (22.4) | 243 (22.0) | 35 (19.6) |  |
| High-school or associate degree | 1700 (39.4) | 784 (37.8) | 392 (40.8) | 452 (40.9) | 72 (40.2) |  |
| Less than High-school | 544 (12.6) | 235 (11.3) | 123 (12.8) | 150 (13.6) | 36 (20.1) |  |
| **Marital status** | |  |  |  |  | 0.063 |
| Married | 2516 (58.3) | 1227 (59.2) | 551 (57.3) | 650 (58.8) | 88 (49.2) |  |
| Unmarried | 1803 (41.7) | 846 (40.8) | 410 (42.7) | 456 (41.2) | 91 (50.8) |  |
| **Residence** | |  |  |  |  | 0.868 |
| Rural | 1349 (31.2) | 636 (30.7) | 301 (31.3) | 354 (32.0) | 58 (32.4) |  |
| Urban | 2970 (68.8) | 1437 (69.3) | 660 (68.7) | 752 (68.0) | 121 (67.6) |  |
| **Total wealth income** | | |  |  |  | <0.001 |
| Highest | 1556 (36.0) | 788 (38.0) | 344 (35.8) | 382 (34.5) | 42 (23.5) |  |
| Quartile 2 | 916 (21.2) | 392 (18.9) | 223 (23.2) | 252 (22.8) | 49 (27.4) |  |
| Quartile 3 | 1396 (32.3) | 697 (33.6) | 287 (29.9) | 357 (32.3) | 55 (30.7) |  |
| Lowest | 451 (10.4) | 196 (9.5) | 107 (11.1) | 115 (10.4) | 33 (18.4) |  |
| **BMI (kg/m^2^)** | |  |  |  |  | <0.001 |
| Underweight | 50 (1.2) | 26 (1.3) | 9 (0.9) | 14 (1.3) | 1 (0.6) |  |
| Normal | 1266 (29.3) | 668 (32.2) | 254 (26.4) | 313 (28.3) | 31 (17.3) |  |
| Obesity | 968 (22.4) | 493 (23.8) | 197 (20.5) | 239 (21.6) | 39 (21.8) |  |
| Overweight | 2035 (47.1) | 886 (42.7) | 501 (52.1) | 540 (48.8) | 108 (60.3) |  |
| **Physical activity** | |  |  |  |  | <0.001 |
| No | 843 (19.5) | 331 (16.0) | 193 (20.1) | 253 (22.9) | 66 (36.9) |  |
| Yes | 3476 (80.5) | 1742 (84.0) | 768 (79.9) | 853 (77.1) | 113 (63.1) |  |
| **Drinking** |  |  |  |  |  | <0.001 |
| No | 1989 (46.1) | 897 (43.3) | 445 (46.3) | 543 (49.1) | 104 (58.1) |  |
| Yes | 2330 (53.9) | 1176 (56.7) | 516 (53.7) | 563 (50.9) | 75 (41.9) |  |
| **Smoking** |  |  |  |  |  | <0.001 |
| No | 1982 (45.9) | 1040 (50.2) | 403 (41.9) | 470 (42.5) | 69 (38.5) |  |
| Yes | 2337 (54.1) | 1033 (49.8) | 558 (58.1) | 636 (57.5) | 110 (61.5) |  |
| **Sleep disturbances** |  |  |  |  |  | <0.001 |
| Low | 3194 (74.0) | 1567 (75.6) | 693 (72.1) | 824 (74.5) | 110 (61.5) |  |
| High | 1125 (26.0) | 506 (24.4) | 268 (27.9) | 282 (25.5) | 69 (38.5) |  |
| **Depressive symptoms** |  |  |  |  |  | <0.001 |
| Low | 3906 (90.4) | 1927 (93.0) | 849 (88.3) | 984 (89.0) | 146 (81.6) |  |
| Increasing | 413 (9.6) | 146 (7.0) | 112 (11.7) | 122 (11.0) | 33 (18.4) |  |
| **ADLs limitations** | |  |  |  |  | <0.001 |
| Low | 4080 (94.5) | 1992 (96.1) | 897 (93.3) | 1031 (93.2) | 160 (89.4) |  |
| Increasing | 239 (5.5) | 81 (3.9) | 64 (6.7) | 75 (6.8) | 19 (10.6) |  |
| **IADLs limitations** | |  |  |  |  | <0.001 |
| Low | 4193 (97.1) | 2027 (97.8) | 935 (97.3) | 1065 (96.3) | 166 (92.7) |  |
| Increasing | 126 (2.9) | 46 (2.2) | 26 (2.7) | 41 (3.7) | 13 (7.3) |  |

Notes: ADLs =activities of daily living. BMI = body mass index. IADLs=instrumental activities of daily living.

Table S6. Trajectories of sleep disturbances, depressive symptoms, ADLs limitations, and IADLs limitations and risk of cognitive impairment

|  | **Model 1** |  | **Model 2** |
| --- | --- | --- | --- |
|  | **HR (95% CI)** |  | **HR (95% CI)** |
| **Sleep disturbances** |  |  |  |
| Low | 1 (reference) |  | 1 (reference) |
| High | 1.01 (0.89, 1.15) |  | 1.01 (0.88, 1.14) |
| **Depressive symptoms** |  |  |  |
| Low | 1 (reference) |  | 1 (reference) |
| Increasing | 1.39 (1.17, 1.65) |  | 1.28 (1.07, 1.53) |
| **ADLs limitations** |  |  |  |
| Low | 1 (reference) |  | 1 (reference) |
| Increasing | 1.13 (0.90, 1.42) |  | 1.11 (0.88, 1.40) |
| **IADLs limitations** |  |  |  |
| Low | 1 (reference) |  | 1 (reference) |
| Increasing | 1.87 (1.42, 2.45) |  | 1.84 (1.40, 2.41) |

Notes: model 1 adjusted for age, gender, educational level, marital status, residence, total wealth income, self-reported body mass index, physical activity, drinking, smoking, all four trajectories, cardiovascular diseases (stroke, hypertension, heart disease); model 2 additionally adjusted other diseases (type 2 diabetes, chronic lung disease, arthritis, psychological diseases and cancer). Data were represented as HR and 95%CI. ADLs =activities of daily living. HR=hazard ratio. IADLs=instrumental activities of daily living. 95%CI=95% confidence interval.

**Table S7. Subgroup analysis of the trajectories of sleep disturbances and risk of cognitive impairment**

| **Subgroup** | **HR (95% CI)** | **P for interaction** |
| --- | --- | --- |
| **Age group (years)** | | 0.438 |
| <70 | 0.77 (0.42, 1.44) | |
| 70~79 | 1.08 (0.92, 1.28) | |
| ≥80 | 0.97 (0.79, 1.20) | |
| **Gender** |  | 0.322 |
| Female | 0.98 (0.84, 1.15) | |
| Male | 1.10 (0.88, 1.37) | |
| **Educational level** | | 0.969 |
| College degree or above | 1.02 (0.73, 1.43) | |
| Some college or associate degree | 0.97 (0.72, 1.29) | |
| High-school or associate degree | 1.04 (0.86, 1.26) | |
| Less than High-school | 1.05 (0.80, 1.37) | |
| **Marital status** | | 0.526 |
| Married | 0.99 (0.82, 1.19) | |
| Unmarried | 1.05 (0.88, 1.25) | |
| **Residence** | | 0.104 |
| Rural | 0.85 (0.67, 1.07) | |
| Urban | 1.10 (0.94, 1.28) | |
| **Total wealth income** | | 0.401 |
| Highest | 0.95 (0.75, 1.19) | |
| Quartile 2 | 0.96 (0.73, 1.25) | |
| Quartile 3 | 1.02 (0.81, 1.28) | |
| Lowest | 1.33 (0.95, 1.86) | |
| **BMI (kg/m^2^)** | | 0.938 |
| Underweight | 1.35 (0.28, 6.55) | |
| Normal | 1.02 (0.81, 1.28) | |
| Obesity | 0.99 (0.76, 1.30) | |
| Overweight | 0.99 (0.82, 1.20) | |
| **Physical activity** | | 0.683 |
| No | 0.98 (0.77, 1.26) | |
| Yes | 1.02 (0.88, 1.18) | |
| **Drinking** |  | 0.569 |
| No | 0.97 (0.82, 1.16) | |
| Yes | 1.08 (0.89, 1.31) | |
| **Smoking** |  | 0.679 |
| No | 0.98 (0.81, 1.18) | |
| Yes | 1.05 (0.88, 1.25) | |
| **Depressive symptoms** | | 0.902 |
| Low | 1.01 (0.88, 1.16) | |
| Increasing | 1.05 (0.77, 1.44) | |
| **Multimorbidity status** | | 0.271 |
| Low | 1.10 (0.91, 1.33) | |
| Increasing early | 1.03 (0.79, 1.35) | |
| Increasing late | 0.82 (0.63, 1.07) | |
| High | 1.26 (0.77, 2.06) | |
| **ADLs limitations** | | 0.383 |
| Low | 1.00 (0.87, 1.14) | |
| Increasing | 1.41 (0.92, 2.17) | |
| **IADLs limitations** | | 0.619 |
| Low | 1.00 (0.88, 1.14) | |
| Increasing | 1.40 (0.79, 2.46) | |

Notes: all models were model 3. Data were represented as HR and 95%CI. ADLs =activities of daily living. BMI = body mass index. HR=hazard ratio. IADLs=instrumental activities of daily living. 95%CI=95% confidence interval.

**Table S8. Subgroup analysis of the trajectories of depressive symptoms and risk of cognitive impairment**

| **Subgroup** | **HR (95% CI)** | **P for interaction** |
| --- | --- | --- |
| **Age group (years)** | | 0.775 |
| <70 | 1.72 (0.84, 3.52) | |
| 70~79 | 1.35 (1.08, 1.69) | |
| ≥80 | 1.36 (1.02, 1.83) | |
| **Gender** |  | 0.688 |
| Female | 1.37 (1.12, 1.67) | |
| Male | 1.49 (1.06, 2.09) | |
| **Educational level** | | 0.745 |
| College degree or above | 1.57 (0.96, 2.58) | |
| Some college or associate degree | 1.59 (1.07, 2.34) | |
| High-school or associate degree | 1.32 (1.01, 1.73) | |
| Less than High-school | 1.24 (0.88, 1.74) | |
| **Marital status** | | 0.76 |
| Married | 1.38 (1.03, 1.86) | |
| Unmarried | 1.40 (1.13, 1.74) | |
| **Residence** | | 0.227 |
| Rural | 1.70 (1.24, 2.33) | |
| Urban | 1.28 (1.04, 1.57) | |
| **Total wealth income** | | 0.156 |
| Highest | 1.56 (1.11, 2.20) | |
| Quartile 2 | 1.06 (0.75, 1.51) | |
| Quartile 3 | 1.55 (1.12, 2.14) | |
| Lowest | 1.54 (1.05, 2.27) | |
| **BMI (kg/m^2^)** | | 0.793 |
| Underweight | 0.47 (0.04, 6.01) | |
| Normal | 1.28 (0.92, 1.79) | |
| Obesity |  | 0.166 |
| Overweight | 1.21 (0.90, 1.63) | |
| **Physical activity** | 1.51 (1.22, 1.87) | |
| No |  | 0.302 |
| Yes | 1.46 (1.18, 1.80) | |
| **Drinking** | 1.22 (0.91, 1.64) | |
| No |  | 0.6 |
| Yes | 1.33 (1.02, 1.73) | |
| **Smoking** | 1.44 (1.15, 1.81) | |
| No |  |  |
| Yes |  |  |
| **Sleep disturbances** |  | 0.902 |
| Low | 1.37 (1.10, 1.72) | |
| High | 1.36 (1.03, 1.78) | |
| **Multimorbidity status** | | 0.596 |
| Low | 1.35 (1.02, 1.80) | |
| Increasing early | 1.66 (1.18, 2.34) | |
| Increasing late | 1.29 (0.93, 1.80) | |
| High | 0.90 (0.48, 1.67) | |
| **ADLs limitations** | | 0.479 |
| Low | 1.34 (1.11, 1.63) | |
| Increasing | 1.59 (1.01, 2.49) | |
| **IADLs limitations** | | 0.759 |
| Low | 1.38 (1.15, 1.65) | |
| Increasing | 1.51 (0.82, 2.78) | |

Notes: all models were model 3. Data were represented as HR and 95%CI. ADLs =activities of daily living. BMI = body mass index. HR=hazard ratio. IADLs=instrumental activities of daily living. 95%CI=95% confidence interval.

**Table S9.** **Subgroup analysis of the trajectories of multimorbidity status and risk of cognitive impairment**

| **Subgroup** | **Increasing early** | **Increasing late** | **High** | **P for interaction** |
| --- | --- | --- | --- | --- |
| **Age group (years)** | |  |  | 0.269 |
| <70 | 1.34 (0.75, 2.42) | 0.82 (0.43, 1.57) | 1.33 (0.37, 4.79) | |
| 70~79 | 0.90 (0.74, 1.10) | 1.03 (0.86, 1.23) | 1.43 (1.05, 1.95) | |
| ≥80 | 0.92 (0.72, 1.17) | 0.78 (0.61, 0.99) | 1.42 (0.94, 2.14) | |
| **Gender** |  |  |  | 0.929 |
| Female | 0.92 (0.77, 1.11) | 0.90 (0.75, 1.08) | 1.51 (1.12, 2.02) | |
| Male | 0.92 (0.73, 1.17) | 0.92 (0.74, 1.15) | 1.31 (0.86, 2.00) | |
| **Educational level** | |  |  | 0.25 |
| College degree or above | 1.18 (0.84, 1.67) | 1.03 (0.74, 1.45) | 1.65 (0.85, 3.20) | |
| Some college or associate degree | 0.86 (0.61, 1.22) | 1.04 (0.76, 1.42) | 0.97 (0.49, 1.94) | |
| High-school or associate degree | 0.92 (0.74, 1.15) | 0.78 (0.62, 0.97) | 1.30 (0.90, 1.88) | |
| Less than High-school | 0.84 (0.60, 1.17) | 1.00 (0.73, 1.36) | 1.93 (1.22, 3.04) | |
| **Marital status** | |  |  | 0.815 |
| Married | 0.98 (0.80, 1.20) | 0.97 (0.80, 1.17) | 1.45 (1.00, 2.09) | |
| Unmarried | 0.88 (0.72, 1.09) | 0.87 (0.71, 1.07) | 1.42 (1.03, 1.95) | |
| **Residence** | |  |  | 0.817 |
| Rural | 0.88 (0.68, 1.15) | 0.92 (0.72, 1.18) | 1.57 (1.05, 2.36) | |
| Urban | 0.95 (0.80, 1.13) | 0.92 (0.77, 1.09) | 1.34 (0.99, 1.81) | |
| **Total wealth income** | | |  | 0.338 |
| Highest | 1.09 (0.84, 1.41) | 0.93 (0.72, 1.20) | 2.04 (1.29, 3.22) | |
| Quartile 2 | 0.93 (0.69, 1.26) | 0.94 (0.71, 1.25) | 1.22 (0.75, 2.00) | |
| Quartile 3 | 0.80 (0.61, 1.05) | 0.90 (0.70, 1.15) | 0.88 (0.52, 1.49) | |
| Lowest | 1.00 (0.67, 1.50) | 0.97 (0.64, 1.47) | 1.86 (1.11, 3.10) | |
| **BMI (kg/m^2^)** | |  |  | 0.775 |
| Underweight | 0.48 (0.07, 3.36) | 0.82 (0.15, 4.39) | 0.00 (0.00, Inf) | |
| Normal | 1.13 (0.87, 1.47) | 0.85 (0.66, 1.09) | 1.46 (0.87, 2.45) | |
| Obesity | 1.00 (0.74, 1.36) | 0.93 (0.70, 1.24) | 1.38 (0.82, 2.30) | |
| Overweight | 0.89 (0.72, 1.11) | 0.97 (0.78, 1.20) | 1.51 (1.09, 2.10) | |
| **Physical activity** | |  |  | 0.926 |
| No | 0.98 (0.72, 1.33) | 1.01 (0.76, 1.34) | 1.60 (1.06, 2.40) | |
| Yes | 0.91 (0.77, 1.08) | 0.89 (0.76, 1.05) | 1.37 (1.01, 1.85) | |
| **Drinking** |  |  |  | 0.322 |
| No | 1.05 (0.87, 1.28) | 0.93 (0.77, 1.13) | 1.52 (1.12, 2.05) | |
| Yes | 0.81 (0.65, 1.01) | 0.92 (0.75, 1.13) | 1.28 (0.84, 1.94) | |
| **Smoking** |  |  |  | 0.653 |
| No | 0.97 (0.78, 1.20) | 0.83 (0.67, 1.03) | 1.37 (0.93, 2.02) | |
| Yes | 0.93 (0.76, 1.13) | 0.99 (0.82, 1.19) | 1.49 (1.09, 2.03) | |
| **Sleep disturbances** |  |  |  | 0.271 |
| Low | 0.95 (0.80, 1.14) | 1.00 (0.85, 1.18) | 1.41 (1.03, 1.93) | |
| High | 0.90 (0.69, 1.18) | 0.75 (0.57, 0.99) | 1.45 (0.99, 2.13) | |
| **Depressive symptoms** |  |  |  | 0.596 |
| Low | 0.90 (0.77, 1.06) | 0.93 (0.80, 1.08) | 1.49 (1.14, 1.95) | |
| Increasing | 1.16 (0.77, 1.75) | 0.93 (0.63, 1.38) | 1.26 (0.72, 2.20) | |
| **ADLs limitations** | |  |  | 0.814 |
| Low | 0.92 (0.79, 1.07) | 0.91 (0.78, 1.05) | 1.46 (1.13, 1.89) | |
| Increasing | 1.23 (0.73, 2.06) | 1.07 (0.62, 1.84) | 1.61 (0.73, 3.53) | |
| **IADLs limitations** | |  |  | 0.401 |
| Low | 0.93 (0.80, 1.08) | 0.90 (0.78, 1.04) | 1.37 (1.06, 1.77) | |
| Increasing | 1.03 (0.50, 2.10) | 0.87 (0.44, 1.72) | 2.77 (1.20, 6.43) | |

Notes: all models were model 3. Data were represented as HR and 95%CI. ADLs =activities of daily living. BMI = body mass index. HR=hazard ratio. IADLs=instrumental activities of daily living. 95%CI=95% confidence interval.

**Table S10. Subgroup analysis of the trajectories of ADLs limitations and risk of cognitive impairment**

| **Subgroup** | **HR (95% CI)** | **P for interaction** |
| --- | --- | --- |
| **Age group (years)** | | 0.439 |
| <70 | 0.94 (0.27, 3.21) | |
| 70~79 | 1.13 (0.83, 1.53) | |
| ≥80 | 1.11 (0.77, 1.60) | |
| **Gender** |  | 0.472 |
| Female | 1.23 (0.94, 1.61) | |
| Male | 0.90 (0.57, 1.42) | |
| **Educational level** | | 0.631 |
| College degree or above | 1.34 (0.72, 2.49) | |
| Some college or associate degree | 1.06 (0.64, 1.76) | |
| High-school or associate degree | 1.25 (0.87, 1.81) | |
| Less than High-school | 0.85 (0.52, 1.37) | |
| **Marital status** | | 0.772 |
| Married | 1.17 (0.80, 1.70) | |
| Unmarried | 1.09 (0.81, 1.47) | |
| **Residence** | | 0.129 |
| Rural | 0.93 (0.57, 1.51) | |
| Urban | 1.18 (0.90, 1.54) | |
| **Total wealth income** | | 0.535 |
| Highest | 1.15 (0.72, 1.83) | |
| Quartile 2 | 0.91 (0.54, 1.54) | |
| Quartile 3 | 1.13 (0.73, 1.74) | |
| Lowest | 1.14 (0.70, 1.86) | |
| **BMI (kg/m^2^)** | | 0.674 |
| Underweight | NA |  |
| Normal | 1.08 (0.67, 1.74) | |
| Obesity | 0.99 (0.55, 1.78) | |
| Overweight | 1.21 (0.89, 1.63) | |
| **Physical activity** | | 0.708 |
| No | 1.00 (0.72, 1.38) | |
| Yes | 1.19 (0.86, 1.65) | |
| **Drinking** |  | 0.56 |
| No | 1.08 (0.80, 1.45) | |
| Yes | 1.20 (0.82, 1.73) | |
| **Smoking** |  | 0.792 |
| No | 1.28 (0.92, 1.77) | |
| Yes | 0.95 (0.69, 1.32) | |
| **Sleep disturbances** |  | 0.383 |
| Low | 1.06 (0.78, 1.44) | |
| High | 1.22 (0.85, 1.75) | |
| **Depressive symptoms** |  | 0.479 |
| Low | 1.05 (0.79, 1.39) | |
| Increasing | 1.15 (0.76, 1.75) | |
| **Multimorbidity status** | | 0.814 |
| Low | 1.05 (0.71, 1.56) | |
| Increasing early | 1.32 (0.85, 2.04) | |
| Increasing late | 1.18 (0.76, 1.84) | |
| High | 0.50 (0.21, 1.18) | |
| **IADLs limitations** | | 0.448 |
| Low | 1.18 (0.91, 1.53) | |
| Increasing | 0.89 (0.52, 1.55) | |

Notes: all models were model 3. Data were represented as HR and 95%CI. ADLs =activities of daily living. BMI = body mass index. HR=hazard ratio. IADLs=instrumental activities of daily living. 95%CI=95% confidence interval.
